# The effect of a one-time mindfulness intervention on body and mind in healthy adolescents using multimodal measurements

**DOI:** 10.3389/fpsyt.2024.1503379

**Published:** 2025-01-23

**Authors:** Angelika Ecker, Charlotte Fritsch, Daniel Schleicher, Ricarda Jacob, Stephanie Kandsperger, Romuald Brunner, Irina Jarvers

**Affiliations:** Department of Child and Adolescent Psychiatry and Psychotherapy, University of Regensburg, Regensburg, Germany

**Keywords:** mindfulness, body scan, state mindfulness, mental burden, heart rate, heart rate variability, adolescents

## Abstract

**Background:**

Mindfulness-based interventions can improve psychological well-being and reduce symptoms of mental burden, including among adolescents. Relationships between basic mindfulness (trait mindfulness) and the immediate effects of a single mindfulness intervention have not been thoroughly researched, especially in adolescents. In this study, we aimed to elucidate these aspects by using a multimodal approach—measuring the effect of a single mindfulness intervention on both subjective and physiological parameters.

**Methods:**

A total of 78 healthy adolescents (12–19 years of age, 50% female) were assigned to either a mindfulness or an active control group. Before and after the interventions, subjective parameters (mood, stress, and state mindfulness) were assessed using bipolar visual analogue scales. Physiological parameters (heart rate and heart rate variability) were measured before and during the interventions. Participants also completed the Self-Compassion Scale (SCS-D) with the subscale “mindfulness” as an assessment of trait mindfulness, and the State-Trait Anxiety-Depression Inventory (STADI).

**Results:**

Our results showed no significant interactions between time and intervention, either subjectively or physiologically. For heart rate, we found a main effect of time. For all subjective parameters, we observed a main effect of trait mindfulness. Age was a relevant factor for heart rate and state mindfulness, suggesting age effects. We also observed strong correlations between trait mindfulness, trait anxiety, and depression scores.

**Conclusion:**

A single mindfulness intervention had no immediate observable effects in our healthy adolescent sample, and possible reasons for this finding are discussed. Nevertheless, the present data show the potential for increased resilience through mindfulness in adolescence.

## 1 Introduction

Among adolescents, high stress levels are linked to increased mental health problems and reduced well-being. Mental health problems affect up to 20% of children and adolescents worldwide (1–4). This alarmingly high rate is particularly worrying due to the prolonged impact into adulthood (4). Therefore, it is of particular interest to strengthen the resilience of youths, to prevent progression to mental health issues at an early stage.

Mindfulness-based interventions (MBIs) have become increasingly popular, especially for promoting psychological well-being and symptom reduction regarding stress or depressive symptoms (5). In a recent review, Porter and colleagues (6) examined 27 studies of children and adolescents using MBIs, and found that most studies showed effects of symptom reduction, e. g. depression symptoms, anxiety and stress. However, these effects were observed over a relatively long-term period, ranging from four weeks to five months (6). While general improvements of mindfulness were observed, the review also highlighted notable methodological inconsistency in the operationalization of mindfulness across studies (6).

When examining mindfulness, it is important to distinguish between mindfulness as a state versus a trait (7). Trait mindfulness refers to an individual’s general tendency to act mindfully in daily life and across various situations (8). Such dispositional mindfulness can be improved by regularly practicing mindfulness exercises over an extended period (7, 9). On the other hand, state mindfulness refers to the level of mindfulness at a given moment, characterized by being attentive and accepting of all present sensations (7). This state can be immediately heightened through mindfulness-related exercises (10). Thus, the improvements attained through MBIs primarily relate to trait mindfulness. An increase of state mindfulness can be measured immediately after a mindfulness intervention—for example, breathing exercises, body scans, attention to movement, and mindful walking, which are basic exercises in mindfulness (11). In a recent study, Sparacio and colleagues (12) reported that the most widely used mindfulness exercise was the body scan, which most effectively reduced stress. Investigations of state mindfulness can help to illuminate the specific psychological and physiological mechanisms involved in mindfulness. Moreover, the application of quick and easy one-time mindfulness exercises is particularly suitable for children and adolescents.

To date, research on the immediate effects of one-time mindfulness exercises has been limited, and mainly confined to the adult domain. One study revealed that a single use of a web-based mindfulness exercise yielded a significantly increased post-intervention mindfulness state in the mindfulness condition, and not in the passive control group (13). Moreover, one-time mindfulness exercises are reportedly effective for reducing induced distress (14), perceived stress, preservative thinking, symptoms of depression, and anxiety, all with small-to-medium effect sizes (15). A meta-analysis by Schumer et al. (16) revealed that a mindfulness practice influenced affect in a manner that did not depend on the intervention’s duration, but these results are based on adult samples. Overall, the available studies indicate that even brief mindfulness exercises can be sufficient to foster a non-judgmental and non-reactive attitude towards occurring events and thoughts, as well as positive effects on well-being.

Fewer studies have examined the effect of a single MBI among children and adolescents, and these studies are often focused on specific topics. For example, Petter and colleagues (17) examined how mindful attention manipulation influenced pain responses in healthy adolescents with different meditation experiences. They found that state mindfulness was related to improved pain reactions, but the mindfulness intervention was only effective among adolescents engaged in regular meditation practice (17), which may reflect the interplay of state and trait mindfulness. Another study investigated the effect of a ten-minute mindfulness practice (stretching and mindful breathing), versus a control condition (quiet play with non-stimulation toys), which revealed no change in self-reported calmness (18). These findings suggest that children and adolescents may experience immediate effects of MBI. However, several questions remain unanswered and require further investigation: can state mindfulness be enhanced in healthy children and adolescents through a single MBI, and if so, in a comparable effect size to adults? What influence does daily meditation practice have for this effect? Are there developmental or gender-related differences? Addressing these gaps will be essential to deepen our understanding of the impact of MBIs on young individuals.

In addition to the subjective effects of MBIs on mood or symptom severity, autonomic nervous system (ANS) changes can also be examined as a physiological indicator of the effects of mindfulness exercises. Such investigations can help uncover the biological mechanisms underlying the effects of MBIs. In particular, parameters of heart rate variability (HRV) seem promising (19). High HRV is associated with a more efficient ANS (i.e., in reaction to stress), which is reportedly affected by MBIs (20). HRV has been used as a short-term indicator of MBI effectiveness—for example, to assess acute cardiovascular effects during each mindfulness session in a study involving ten days of mindfulness practice, which resulted in higher HRV compared to a passive control group (21). When examining an even shorter time period (i.e., a single session), HRV was slightly improved after mindfulness-based cognitive training in an adolescent sample with attention-deficit/hyperactivity disorder compared to a control condition (22). As another parameter of the ANS, heart rate (HR) has also been investigated in MBI studies, with decreased HR indicating a relaxing effect of MBIs (23). Thus, ANS parameters—primarily HRV and HR—have a strong ability to reflect the effect of MBIs on physiological processes, and can be easily and non-invasively assessed. Studies in which the multimodal approach has proven effective for investigating the effects of mindfulness induction on both subjective and objective outcomes in adolescents include those conducted after a psychosocial stress induction, for example after a psychosocial stress induction (24, 25).

Overall, there remain uncertainties in samples of healthy adolescents regarding the strength of the association between state and trait mindfulness, the effectiveness of a single mindfulness intervention, and the transferability of prior findings and interventions to adolescents. There exists a need for a comprehensive evaluation of the multimodal (subjective and objective) effects of single mindfulness interventions. Therefore, in the present study, we aimed to investigate whether a single MBI has positive effects on healthy adolescents, with specific focus on subjective measures of well-being and state mindfulness, as well as on objective measures of HRV and HR. We additionally aimed to explore the role of trait mindfulness in these effects. We expect that the mindfulness intervention will result in increased subjective well-being, higher HRV, and reduced HR, more so than in the control condition. Additionally, we anticipate that trait mindfulness may play an important role in these mechanisms.

## 2 Methods

### 2.1 Design

The study was conducted as a 2 × 2 design. The between-subjects factor was group: mindfulness-based intervention (MBI) vs. active control group. The within-subjects factor was time: pre-intervention vs. post-intervention. We conducted an a-priori power analysis to determine the required sample size. For a desired power of 95%, and an expected mean effect size of *f* = 0.31 (13), a sample size of *N* = 36 participants (*n* = 18 per condition) was estimated to be sufficient. Since Mahmood et al. (13), was the first study to investigate the immediate effect of a single mindfulness intervention, the effect sizes were based on an adult sample (mean age: 33.56 years). The first half of participants (*n* = 39) were randomly assigned to one of the groups, and later participants were matched according to age and sex, until achieving the final sample size in each group. Participants and their parents or legal guardians were blinded to group assignment.

### 2.2 Sample

The study included *N* = 78 participants. The average age was 15.33 years (*SD* = 2.41), and 50% were female. **Table 1** presents a detailed overview of demographic variables. Inclusion criteria were age between 12–19 years, and sufficient understanding of the German language. Exclusion criteria were past or current psychiatric, psychotherapeutic, or neurological treatments; pregnancy; breastfeeding; intellectual impairment; or attendance at a special school. Recruitment was carried out using e-mail distribution lists, social media accounts, and flyers. This study was approved by the Ethics Committee of the University of Regensburg (No.: 20–2095–101). All participants and their legal guardians gave written informed consent. Participants received a gift voucher worth €25 for their participation.

**Table 1:** Demographic and psychometric characteristics and group comparisons.

	Total sample	Group		Group comparisons
		Mindfulness	Control	
**Number of participants**	*N* = 78	*n* = 40	*n* = 38	
Age in years				
*M (SD)*	15.33 (2.41)	15.40 (2.43)	15.26 (2.41)	*t*(75.8) = 0.25, *p* = .804^a^ TOST: *p*s >.124^b^
Range	12–19	12–19	12–19	
Sex				
Female (%)	39 (50.0)	19 (47.5)	20 (52.6)	*t*(75.8) = 0.45, *p* = .656^a^ TOST: *ps* <.001^b^
Male (%)	39 (50.0)	21 (52.5)	18 (47.4)	
School type				
Mittelschule (%)	4 (5.1)	3 (7.5)	1 (2.6)	*Z* = −0.49, *p* = .621^c^
Realschule (%)	14 (17.9)	7 (17.5)	7 (18.4)	
Gymnasium (%)	39 (50.0)	18 (45.0)	21 (55.3)	
FOS/BOS (%)	5 (6.4)	4 (10.0)	1 (2.6)	
University (%)	11 (14.1)	6 (15.0)	5 (13.2)	
Other (%)	3 (3.8)	–	3 (7.9)	
Missing information (%)	2 (2.6)	2 (5.0)	–	
Mindfulness experience				
No (%)	34 (43.6)	19 (47.5)	15 (39.5)	*Z* = −0.55, *p* = .585^c^
A little (%)	16 (20.5)	7 (17.5)	9 (23.7)	
Some (%)	19 (24.4)	7 (17.5)	12 (31.6)	
Much (%)	5 (6.4)	4 (10.0)	1 (2.6)	
Very much (%)	2 (2.6)	1 (2.5)	1 (2.6)	
Missing information (%)	2 (2.6)	2 (5.0)	–	
Anxiety and Depression				
*M (SD)*	38.31 (10.83)	40.10 (11.09)	36.42 (10.35)	*t*(75.98) = 1.52, *p* = .134^a^
Range	23–66	24–66	23–61	
Trait Mindfulness				
*M (SD)*	13.5 (3.02)	12.95 (3.27)	14.11 (2.65)	*t*(74.2) = −1.72, *p* = .090^a^
Range	6–20	6–20	8–18	

Sex: all participants were asked about sex and gender, which were congruent in all cases. School types: secondary schools following elementary school in Germany; Mittelschule: 9 years of elementary school; Realschule: intermediate level of secondary school, regular duration of 6 years; FOS (Fachoberschule)/BOS (Berufsoberschule): tertiary school to achieve advanced technical college certificate, subject-related entrance qualification or general qualification for university entrance after visiting Realschule, duration: 2–3 years additionally beyond the duration of Realschule; Gymnasium: highest level of secondary school, regular duration of 8–9 years, qualification: general qualification for university entrance. Mindfulness experience was assessed using one item, examples of mindfulness exercises were yoga or meditation. Anxiety and Depression was assessed with via STADI trait. Trait Mindfulness was assessed via SCS-D, subscale mindfulness. ^a^ *t*-test for independent samples, ^b^Welch’s *t*-test using the TOST equivalence test method, ^c^Mann-Whitney-*U*-test.

### 2.3 Materials

#### 2.3.1 Body scan

For the MBI condition, the body scan was selected as the mindfulness exercise because it is categorized as a basic mindfulness exercise (9), and has been associated with the best stress reduction (12), and is thus highly suitable for preventative approaches. In our study, the participants were instructed to consciously be aware of individual areas of their body, to accept all sensations and feelings and not to judge them. If their attention wandered, participants were asked to lead it back to the task, with a non-judgmental attitude (26). To increase the standardization of the procedure, each participant performed the body scan under audio guidance. The duration of the body scan audio was approximately 10 min.

#### 2.3.2 Active control group

To compare the mindfulness intervention with an active control group, we utilized an audiobook excerpt from “Mary Poppins comes back”, of the same duration as the body scan audio guidance. Participants were instructed to listen attentively, as they would in the mindfulness intervention, but without explicitly focusing on mindfulness and awareness of their own physical sensations. While the sensory input was similar between the two conditions, the input explicitly differed in the construct of mindfulness being examined.

### 2.4 Measures

#### 2.4.1 Questionnaires

##### 2.4.1.1 Subjective assessment

Visual analogue scales were utilized to measure the immediate subjective effect before and after the interventions. The three areas examined were mood, stress, and mindfulness (state). Formulations of the scales were adapted from the German “Der Mehrdimensionale Befindlichkeitsfragebogen” [multidimensional mood questionnaire] (MDBF) (27). Participants were asked “How do you feel right now?”, and answered using the following eight 11-point bipolar scales: *mood*, “bad to good” and “tired to awake”; *stress*, “stressed to calm” and “tensed to relaxed”; *mindfulness state*, “critical and judgmental towards myself to accepting myself”, “critical and judgmental towards my environment to accepting my environment”, “unfocused to concentrated”, and “distracted to being in the present moment”. Thus, according to the two-dimensional definition of mindfulness (10), all basic facets of mindfulness were assessed using a small number of items. All items were combined into a mean score according to their respective scale, with scores ≥6 indicating positive well-being, and those <6 indicating a lack of well-being.

##### 2.4.1.2 Trait mindfulness

To assess the participants’ general mindfulness (trait), we used the mindfulness subscale from the Self-Compassion Scale (28), in its German version (SCS-D) (29). This questionnaire comprises 26 items scored on a 5-point Likert scale, ranging from 1 (very rarely) to 5 (very often). A total score can be calculated from the six subscales: mindfulness, common humanity, self-judgment, isolation, over-identification, and self-kindness, with higher scores indicating higher self-compassion. The subscale mindfulness score has shown nearly acceptable internal consistency (Cronbach’s α = .66) and confirmed validity (29).

##### 2.4.1.3 Anxiety and depression

The State-Trait Anxiety-Depression Inventory (STADI) (30) was used to assess anxiety and depression scores, as an indicator of the absence of well-being/mental burden. This questionnaire comprises two subscales (anxiety and depression) as state and trait. In total, the STADI contains 40 items, which are answered using a 4-point Likert scale. Trait item responses range from 1 (almost never) to 4 (almost always), and state item responses from 1 (not at all) to 4 (very). The trait scale was used to evaluate the participants’ mental burden. The anxiety and depression scales exhibit reliability within an appropriate range (α = 0.87–0.90). Validity testing has confirmed the convergent and discriminant correlations, as well as the factorial validity of the questionnaire (30).

#### 2.4.2 Heart rate and heart rate variability

HR and HRV were measured using the wireless sensor EcgMove 4 (movisens GmbH, Karlsruhe, Germany), which was attached to the participants’ chest using patches. Raw data were preprocessed using the software “DataAnalyzer” (version 1.13.5; movisens, Munich, Germany). As a parameter of HRV, we selected the root mean square of successive differences (RMSSD) as a time-specific marker, which is particularly suitable for short-term changes (31). We also included the frequency-based parameters low frequency (LF) and high frequency (HF), and the LF/HF ratio, which are especially notable as indicators of relaxation (32–34). HR and HRV were assessed before the intervention (baseline measurement) and throughout the complete intervention. For the analyses, we selected time-points from the end of baseline (second minute) and at the final third of the intervention (tenth minute) for comparison. This intervention interval was chosen because mindfulness exercises typically end with a return of one’s attention to the surroundings, and small body movements.

### 2.5 Procedure

Participants and their accompanying parent or legal guardian were provided detailed information about the study, and gave their informed consent. Subsequent testing occurred without the presence of the accompanying person. At the beginning of the examination, the participants themselves attached the wireless sensor. Next, the participants used a laptop to digitally provide demographic information and complete questionnaires: SCS-D, STADI, and subjective well-being (pre-intervention). Afterwards, participants were instructed to lay comfortably on their backs on a provided mattress, and were given the option of closing their eyes, while listening to the audio file via headphones. They were asked to attentively follow the audio file, and the implemented instructions, when required. After the baseline and intervention, all participants again answered the subjective well-being questions (post-intervention). Finally, participants were informed about the scope of the study, were invited to give feedback or ask further questions about the study, and removed the attached sensor. Upon completion, participants were given a voucher worth 25€.

### 2.6 Statistical analysis

Group differences in demographic variables were examined using *t*-tests, Welch’s *t*-test, Mann-Whitney *U*-tests, and Two One-Sided Tests (TOST) for equivalence analysis. Bivariate correlations were evaluated using Kendall’s τ. Possible changes due to the intervention were investigated by within-subject comparisons of a two-factor analysis of variance (ANOVA) with repeated measures (pre/post) of subjective scores (mood, stress, and state mindfulness), as well as changes in HR and HRV. To examine the effect of the mindfulness exercise compared to the control intervention, condition was included in the analysis as a between-subject factor, to investigate an interaction effect between time and condition. The analyses also included the following covariates: sex, age, mindfulness trait (subscale “mindfulness” from SCS-D), and experience with mindfulness exercises. Mental burden (STADI trait) was not included as a covariate, due to its high inter-correlation with the mindfulness trait. Exploratory analyses revealed comparable results when including mental burden instead of mindfulness trait. Due to violation of the normal distribution assumption, all HRV parameters and STADI scores were log-transformed regarding their positive skewness, while subjective answers were log-transformed regarding their negative skewness, to achieve the best approximation of a normal distribution. The partial eta-square (η_p_^2^) was calculated as a measure of effect size, with 0.01 considered a small effect, 0.06 a medium effect, and 0.14 a large effect. Statistical analyses were performed using SPSS Statistics 29 software. Equivalence tests were conducted using the TOSTER 0.4.0 module in jamovi version 2.3.28 for Windows. The significance level was set as α = 0.05.

Data regarding the physiological variables were available for only a part of the surveyed sample, due to technical difficulties in the measurements, likely related to the supine position of the participants. Heart rate was available for *n* = 62, and heart rate variability for *n* = 56. Due to a technical issue with the survey platform, one participant could not complete the SCS-D and STADI questionnaires, and three participants had to complete “paper and pencil” versions, which were missing questions regarding school type and mindfulness experience. No other technical difficulties occurred.

## 3 Results

**Table 1** presents the demographic information for the study sample, according to group, revealing no significant group differences in age, sex, school type, or prior mindfulness experience. Regarding psychometric characteristics, the groups did not differ significantly in trait mindfulness (subscale mindfulness SCS-D, *t*(74.21) = −1.72, *p* = .090) or in anxiety or depression scores (STADI state: *t*(66.53) = 1.62, *p* = .111; trait: *t*(75.98) = 1.52, *p* = .134). These similarities indicated that the groups can be compared without restriction. Trait mindfulness and trait anxiety and depression scores showed a medium inter-correlation (subscale mindfulness SCS-D× STADI Trait: τ = −0.34, *p* <.001); therefore, they were not considered together in the following models. Rather, the analysis focused on trait mindfulness, in line with the research question. However, for exploratory purposes, each model was also tested with trait anxiety and depression, and these results did not differ from those obtained using trait mindfulness.

**Figure 1:**
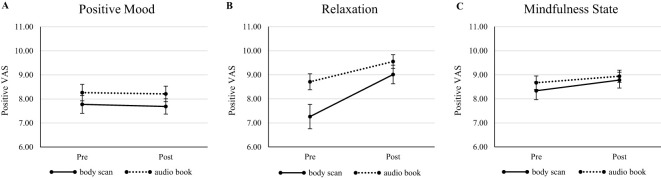
Courses of subjective parameters pre-intervention to post-intervention regarding **(A)** Positive Mood; **(B)** Relaxation; and **(C)** Mindfulness State. VAS, visual analogue scale [1;11], bipolar scale, meaning values <6 represent the negative pole, and ≥6 represent the positive pole. Since all values ranged in positive pole, only positive VAS are depicted. Error bars show standard error.

**Figure 2:**
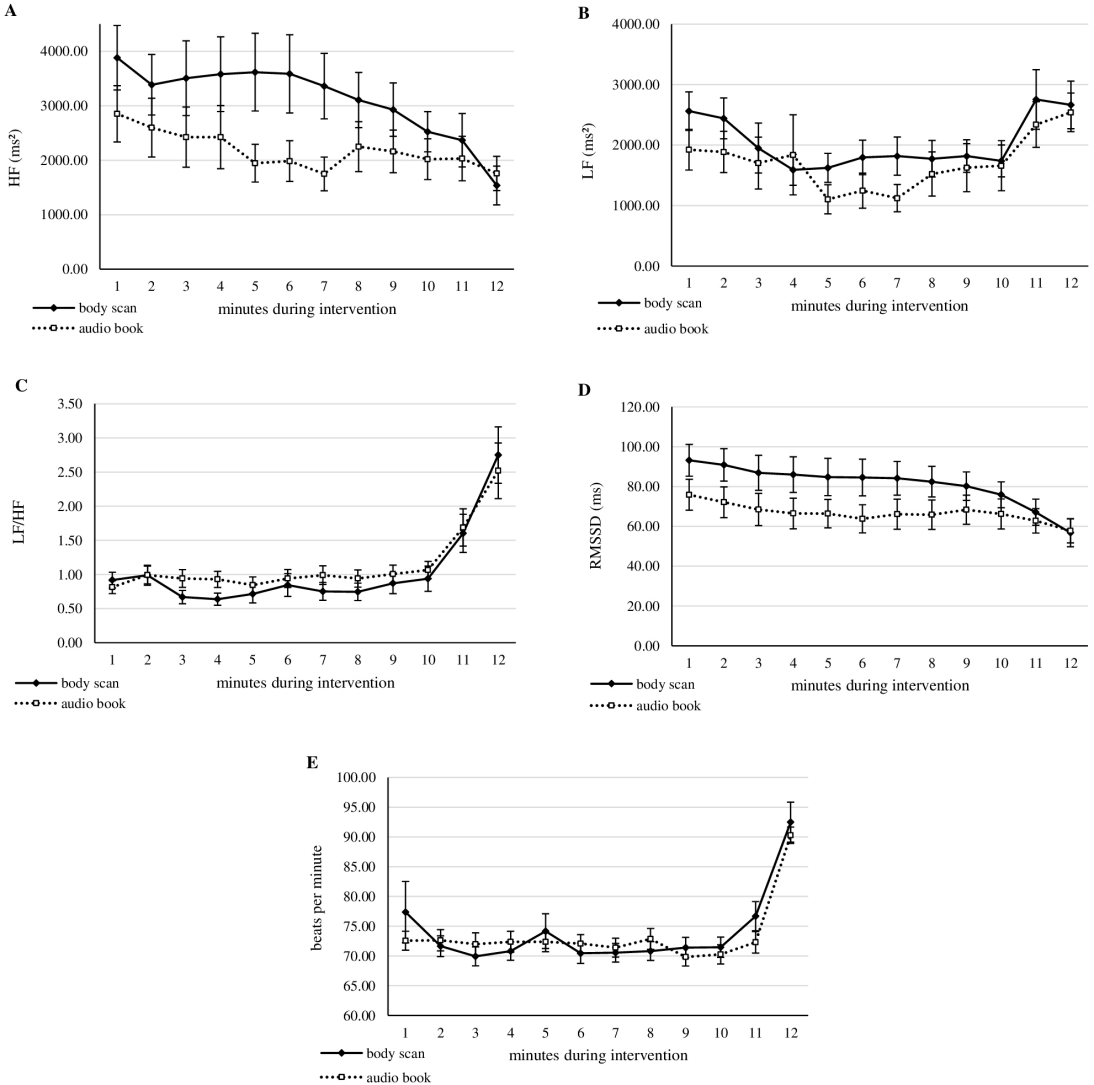
Courses of physiological parameters during intervention. For all courses, the first 2 minutes cover the baseline measurement, minutes 3–12 cover the intervention, while body movements were possible during the 2 last minutes of intervention. **(A–D)** The parameter of heart rate variability. HF, high frequency; LF, low frequency; LF/HF, ratio of low to high frequency; RMSSD, root mean square of successive differences. **(E)** Heart rate. Error bars show standard error.

The courses of the parameters during the intervention (MBI or active control) are presented in **Figure 1** (subjective data) and **Figure 2** (physiological data). **Table 2** shows the results of the ANOVAs on the various variables. Overall, none of the investigated parameters exhibited the expected effect of the mindfulness intervention (time × condition). Nevertheless, the analysis revealed interesting results. All subjective measures (mood, stress, and state mindfulness) showed a main effect for trait mindfulness, with positive correlations in the subsequent analysis of the direction for all post measurements (τ = [0.18; 0.30], all *p* <.026), suggesting that individuals with higher trait mindfulness also experienced feeling better, more calmness and greater state mindfulness, especially after the intervention across both conditions. Additionally, for state stress and state mindfulness, age was a significant covariate. In state mindfulness, lower age was linked to higher state mindfulness values, except among 18-year-olds. In state stress, no clear trend was detectable. For the physiological parameters, mixed results were found. HF showed a main effect for trait mindfulness (suggesting higher HF values with higher trait mindfulness values) and a main effect of condition, revealing a general effect observable in the descriptive data: participants in the MBI condition showed higher HF values than participants of the active control group, before as well as after the intervention. LF and LF/HF both exhibited a significant effect of sex (females < males), while LF/HF also exhibited a main effect of trait mindfulness, similar to HF, but suggesting an inverse relationship (higher trait mindfulness associated with lower LF/HF). On the other hand, RMSSD showed a main effect for time, with pre-intervention values being higher than post-intervention values. Additionally, the RMSSD showed an effect of condition, with MBI group showing higher RMSSD values than the active control group. Lastly, for HR, we observed an effect of time (pre-intervention > post-intervention) and an interaction effect of time × age. The courses between pre-intervention and post-intervention diverged from the age of 16. Starting at this age, we observed a descriptive effect of the interventions, namely a lower heart rate after the intervention than before. In contrast, participants of 13–15 years old exhibited barely any differences between pre-intervention and post-intervention.

**Table 2:** ANOVAs regarding psychological and physiological parameters.

	*SS*	*df*	*MS*	*F*	*p*	η_p_^2^
ANOVA subjective mood						
Time	0.14	1	0.14	1.38	.244	0.02
Time*Age	0.02	1	0.02	0.16	.693	<0.01
Time*M_Experience	0.03	1	0.03	0.32	.574	<0.01
Time*T_Mindfulness	0.06	1	0.06	0.61	.437	0.01
Time*Condition	<0.01	1	0.00	0.02	.878	<0.01
Time*Sex	0.04	1	0.04	0.40	.532	0.01
Time*Condition*Sex	0.06	1	0.06	0.57	.454	0.01
Error	7.10	69	0.10			
Age	2.28	1	2.28	3.75	.057	0.05
M_Experience	0.05	1	0.05	0.08	.772	<0.01
**T_Mindfulness**	**4.09**	**1**	**4.09**	**6.71**	**.012**	**0.09**
Condition	0.16	1	0.16	0.25	.615	<0.01
Sex	0.77	1	0.77	1.26	.265	0.02
Condition*Sex	0.20	1	0.20	0.33	.569	<0.01
ANOVA subjective stress						
Time	0.04	1	0.04	0.22	.637	<0.01
Time*Age	<0.01	1	<0.01	0.01	.910	<0.01
Time*M_Experience	0.04	1	0.04	0.23	.632	<0.01
Time*T_Mindfulness	0.06	1	0.06	0.35	.556	0.01
Time*Condition	0.42	1	0.42	2.50	.119	0.03
Time*Sex	0.46	1	0.46	2.72	.104	0.04
Time*Condition*Sex	0.03	1	0.03	0.17	.677	<0.01
Error	11.663	69	0.17			
**Age**	**4.37**	**1**	**4.37**	**5.58**	**.021**	**0.07**
M_Experience	0.78	1	0.78	0.99	.322	0.01
**T_Mindfulness**	**16.22**	**1**	**16.22**	**20.73**	**<.001**	**0.23**
Condition	0.01	1	0.01	0.01	.904	<0.01
Sex	0.77	1	0.77	0.98	.326	0.01
Condition*Sex	0.40	1	0.40	0.52	.475	0.01
ANOVA subjective mindfulness						
Time	0.01	1	0.01	0.16	.691	<0.01
Time*Age	0.06	1	0.06	0.83	.364	0.01
Time*M_Experience	0.08	1	0.08	1.07	.304	0.02
Time*T_Mindfulness	0.06	1	0.06	0.79	.376	0.01
Time*Condition	0.07	1	0.07	0.99	.323	0.01
Time*Sex	0.12	1	0.12	1.64	.204	0.02
Time*Condition*Sex	<0.01	1	<0.01	0.07	.795	<0.01
Error	5.02	69	0.07			
**Age**	**5.76**	**1**	**5.76**	**10.02**	**.002**	**0.13**
M_Experience	0.01	1	0.01	0.02	.893	<0.01
**T_Mindfulness**	**6.54**	**1**	**6.54**	**11.37**	**.001**	**0.14**
Condition	0.33	1	0.33	0.57	.452	0.01
Sex	0.64	1	0.64	1.11	.296	0.02
Condition*Sex	0.16	1	0.16	0.27	.602	<0.01
ANOVA HRV HF						
Time	0.38	1	0.38	2.41	.128	0.05
Time*Age	0.16	1	0.16	1.05	.310	0.02
Time*M_Experience	0.14	1	0.14	0.89	.350	0.02
Time*T_Mindfulness	0.03	1	0.03	0.20	.660	<0.01
Time*Condition	<0.01	1	<0.01	0.02	.898	<0.01
Time*Sex	0.34	1	0.34	2.21	.144	0.05
Time*Condition*Sex	0.11	1	0.11	0.67	.416	0.01
Error	7.17	46	0.16			
Age	5.32	1	5.32	3.14	.083	0.06
M_Experience	1.10	1	1.10	0.65	.426	0.01
**T_Mindfulness**	**7.74**	**1**	**7.74**	**4.56**	**.038**	**0.09**
**Condition**	**11.35**	**1**	**11.35**	**6.70**	**.013**	**0.13**
Sex	0.89	1	0.89	0.53	.472	0.01
Condition*Sex	0.08	1	0.08	0.05	.830	<0.01
ANOVA HRV LF						
Time	0.05	1	0.05	0.13	.720	<0.01
Time*Age	<0.01	1	<0.01	0.01	.913	<0.01
Time*M_Experience	0.64	1	0.64	1.60	.212	0.03
Time*T_Mindfulness	0.22	1	0.22	0.56	.459	0.01
Time*Condition	0.72	1	0.72	1.81	.185	0.04
Time*Sex	0.21	1	0.21	0.53	.469	0.01
Time*Condition*Sex	0.10	1	0.10	0.26	.611	0.01
Error	18.29	46	0.40			
Age	0.72	1	0.72	0.50	.485	0.01
M_Experience	0.17	1	0.17	0.12	.731	<0.01
T_Mindfulness	1.37	1	1.37	0.95	.335	0.02
Condition	4.55	1	4.55	3.15	.082	0.06
**Sex**	**6.44**	**1**	**6.44**	**4.46**	**.040**	**0.09**
Condition*Sex	0.78	1	0.78	0.54	.465	0.01
ANOVA HRV R HF/LF						
Time	0.71	1	0.71	2.33	.134	0.05
Time*Age	0.15	1	0.15	0.48	.494	0.01
Time*M_Experience	0.19	1	0.19	0.62	.435	0.01
Time*T_Mindfulness	0.35	1	0.35	1.14	.292	0.02
Time*Condition	0.53	1	0.53	1.73	.195	0.04
Time*Sex	0.01	1	0.01	0.03	.874	<0.01
Time*Condition*Sex	0.37	1	0.37	1.20	.279	0.03
Error	14.07	46	0.31			
Age	2.31	1	2.31	3.38	.073	0.07
M_Experience	0.37	1	0.37	0.54	.466	0.01
**T_Mindfulness**	**2.81**	**1**	**2.81**	**4.11**	**.049**	**0.08**
Condition	1.66	1	1.66	2.43	.126	0.05
**Sex**	**11.67**	**1**	**11.67**	**17.07**	**<.001**	**0.27**
Condition*Sex	0.32	1	0.32	0.47	.498	0.01
ANOVA HRV RMSSD						
**Time**	**0.12**	**1**	**0.12**	**5.00**	**.030**	**0.09**
Time*Age	0.02	1	0.02	0.94	.338	0.02
Time*M_Experience	0.08	1	0.08	3.25	.078	0.06
Time*T_Mindfulness	0.05	1	0.05	1.97	.167	0.04
Time*Condition	<0.01	1	<0.01	0.20	.659	<0.01
Time*Sex	0.04	1	0.04	1.71	.198	0.03
Time*Condition*Sex	0.04	1	0.04	1.70	.199	0.03
Error	1.16	48	0.02			
Age	1.13	1	1.13	2.26	.139	0.04
M_Experience	1.13	1	1.13	2.27	.139	0.05
T_Mindfulness	1.22	1	1.22	2.45	.124	0.05
**Condition**	**2.81**	**1**	**2.81**	**5.63**	**.022**	**0.10**
Sex	0.22	1	0.22	0.44	.512	0.01
Condition*Sex	0.11	1	0.11	0.22	.644	<0.01
ANOVA HR						
**Time**	**43.86**	**1**	**43.86**	**6.51**	**.014**	**0.11**
Time*Age	**65.98**	**1**	**65.98**	**9.80**	**.003**	**0.15**
Time*M_Experience	13.03	1	13.03	1.93	.170	0.03
Time*T_Mindfulness	0.01	1	0.01	<0.01	.975	<0.01
Time*Condition	2.06	1	2.06	0.31	.582	0.01
Time*Sex	0.49	1	0.49	0.07	.789	<0.01
Time*Condition*Sex	10.86	1	10.86	1.61	.210	0.03
Error	363.71	54	6.74			
Age	11.10	1	11.10	0.06	.801	<0.01
M_Experience	104.01	1	104.01	0.60	.442	0.01
T_Mindfulness	619.44	1	619.44	3.58	.064	0.06
Condition	27.93	1	27.93	0.16	.689	<0.01
Sex	2.93	1	2.93	0.02	.897	<0.01
Condition*Sex	78.86	1	78.86	0.46	.502	0.01

SS, Type III Sum of Squares; df, degrees of freedom; MS, mean square; η_p_^2^, Partial eta Square; M_Experience, Experience with mindfulness exercises [no; very much]; T_Mindfulness, Trait Mindfulness, assessed with a questionnaire (Self-Compassion Scale, subscale mindfulness); Condition, Mindfulness exercise or active control group. Significant effects are highlighted in bold font.

## 4 Discussion

In the present study, we used multimodal measurements (i.e., subjective and physiological indicators) to investigate the effects of a single-session mindfulness intervention, and compared it to an active control group. A total of 78 adolescent participants were divided into two groups, and we assessed their subjective mood, stress, and state mindfulness before and after the intervention. We also recorded and analyzed the physiological parameters of heart rate and heart rate variability. Our analysis considered demographic characteristics, including sex and age, as well as potentially influencing factors, such as mindfulness trait, experience with mindfulness exercises, and general levels of anxiety and depression.

Our results did not show the expected effect that only the group participating in the mindfulness intervention would exhibit improvements of subjective well-being, state mindfulness, HR, and HRV. The only notable change was a HR reduction after the intervention compared to baseline; however, this effect was observed across both groups, not exclusively in the mindfulness group. Thus, listening to an audiobook was as effective for reducing HR as following a guided body scan, which is consistent with the findings of prior studies (35–37). One possible explanation may be the similar attentional processes required for both the body scan and the active control task (38). Our results also revealed an effect of age for HR: older participants benefited from the interventions, whereas younger participants showed little change in HR throughout the interventions. This could reflect an actual developmental effect based on age, or could indicate problems with the intervention for younger participants, e.g., a lack of age-appropriate instruction (6). However, the latter explanation is unlikely because we paid much attention to age-appropriate instruction during the design of the study. In further studies, it would be interesting to investigate whether another age limit can be found, above which a single intervention with attention control has an effect on HR. Furthermore, in both conditions, one must consider the effect of the supine position, which alone can lead to a decrease in HR. It could be helpful to also compare different positions during the exercises in future studies.

While previous research has shown positive outcomes on HRV due to brief mindfulness interventions (21), our present HRV results showed a very mixed, inconsistent and, in some cases, counterintuitive picture. We did not observe any changes over time in the frequency-based parameters of HRV. We found effects of trait mindfulness for HRV HF and the ratio of high and low frequency, but with inconsistent trends, suggesting trait mindfulness as a potentially relevant factor which needs extended attention in the future. Moreover, in contrast to prior findings (21), the time-based parameter RMSSD showed higher values before interventions than after, indicating an increase of stress rather than a decrease, since lower RMSSD values indicate stress (39). Further studies are needed to investigate whether the testing situation was perceived as stressful by participants, or if other mechanisms underlie these results. We observed group differences in the HR and HRV levels—with higher HR, indicating more stress, and higher HRV, indicating less stress, even before the interventions. This finding cannot be fully explained, but it may have contributed to the failure to detect the expected effects, as seen in previous studies. Overall, while the presently reported results regarding the physiological effects of a single mindfulness intervention are interesting, they should be carefully interpreted.

Similar to the physiological parameters, the subjective measures did not indicate any immediate effect of the (mindfulness) intervention. One possible explanation could be that the participants’ subjective responses were already in a positive range before the interventions, potentially leading to a ceiling effect that limited the possibility for further improvement. Another possible explanation might be that youths do not subjectively benefit from a single mindfulness intervention, as suggested by prior research (13, 40). Notably, the assessed domains of subjective well-being (mood and stress/relaxation) and state mindfulness showed positive correlations with trait mindfulness—with higher trait mindfulness being associated with more positive mood, lower stress/higher relaxation, and higher state mindfulness, each with a large effect. While our results did not demonstrate the effect of a single mindfulness intervention, they did indicate the interplay between psychological well-being and a generally mindful attitude in an adolescent sample. Consistently, we found a strong negative association between trait mindfulness and trait anxiety and depression scores—with a higher generally mindful attitude being correlated with lower levels of anxiety and depression in our sample. This finding is in line with previous results (7, 41, 42), and highlights that even in this young age group, trait mindfulness could constitute a factor supporting resilience against common mental health challenges (43). Moreover, the data support the relevance of trait mindfulness, as it was correlated with both anxiety and stress in our study, making its connection to the improvements seen in MBI programs among adolescents particularly evident (44, 45). It is also interesting that our results only showed an effect of age for state stress and state mindfulness as subjective parameters. This could be a development-specific effect (6), in which younger individuals may exhibit higher levels of state mindfulness and, therefore, greater resilience. Further studies are needed to investigate whether this is the explanation, or if these findings result from an age-related bias in ratings. In any case, age is an aspect that should definitely be considered in studies involving adolescents, as specific characteristics have been found, both physiologically and subjectively. Additionally, age has been found to be a moderator for trait mindfulness in a prior study, i.e. regarding dispositional mindfulness and ostracism—the social exclusion or rejection by others. In this context, higher age was more beneficial regarding trait mindfulness (46). This demonstrates the potential of trait mindfulness throughout development and suggests that focusing on it earlier could enhance resilience. The present findings should be interpreted with caution since there are currently very few studies of a single mindfulness exercise in a healthy adolescent population. Notably, single mindfulness interventions seem to particularly affect attention mechanisms, whereas longer training periods are required to affect the usual subjective and physiological outcomes (47), and even then only with small effects (48). Further research in this age group, possibly with different mindfulness exercises and an additional passive control group, could provide interesting insights.

The limitations of this study must be considered when interpreting the results. Notably, we examined only one type of mindfulness exercise: the body scan. Therefore, we cannot make generalizations regarding the lack of effect of a single mindfulness intervention among healthy adolescents, and further investigation is required. However, the lack of effect, despite the body scan being considered the most promising mindfulness exercise (12), is not encouraging for other interventions. Another limitation of this study is the potential for undetected mental health issues among participants. We relied on self-reported data regarding previous psychological treatments and the absence of high emotional distress or mental illnesses. However, we believe that such cases are likely rare and would not significantly impact our findings given our sample size. Additionally, this study did not include a passive control group. Another notable limiting aspect is the assessment of subjective parameters. The categories were surveyed using two or four items, meaning that the reliability of the survey was not optimal. However, this approach was chosen to pragmatically survey the specific constructs of interest. Moreover, an established measurement instrument, the MBFB (27), was used to ensure validity. Future studies should critically examine this aspect.

The present study also has several strengths that must be highlighted. The study included a large well-characterized sample of healthy adolescents, and various important control variables, i.e., the assessment of mental burden and trait mindfulness. The selected age range covers the early and middle puberty age groups, which Porter and colleagues (6) have defined as interesting in this context, due to the developmental trajectories and socioemotional skills of children and adolescents. Additionally, the demographic and psychometric characteristics did not differ between the groups, enabling unrestricted interpretations of the results. Although we conducted an a-priori power analysis, a pre-intervention condition effect was identified upon reaching the required sample size. Therefore, the sample size was doubled to account for missing HR and HRV values and to enable the detection of even smaller effects in adolescents, with effects observed up to an eta of 0.04 with a power of 80%. This adjustment was made to be able to provide the most conclusive results possible. Another strength of this study is our multimodal assessment of the effects of a mindfulness intervention. In addition to subjective questions, which can be prone to bias, we also assessed objective physiological parameters, i.e., HR and various HRV parameters. This combination of subjective and objective measures provides a more comprehensive overview.

## 5 Conclusion

In summary, our present results indicated that a single mindfulness exercise did not improve subjective well-being, state mindfulness, or physiological parameters in healthy adolescents aged 12–19, compared to an active control group. Although previous studies have observed immediate effects of such interventions in adults, this does not yet appear to occur in adolescents. Further research is needed to investigate the underlying mechanisms. A generally mindful attitude is associated with lower mental burden in healthy adolescents, and thus represents a resilience factor for greater well-being.

## Data Availability

The raw data supporting the conclusions of this article will be made available by the authors upon reasonable request.
